# Morphology and Transport Study of Acid–Base Blend Proton Exchange Membranes by Molecular Simulations: Case of Chitosan/Nafion

**DOI:** 10.1021/acs.jpcb.3c05332

**Published:** 2023-12-01

**Authors:** Ehsan Hemmasi, Mahdi Tohidian, Hesam Makki

**Affiliations:** † Department of Polymer and Color Engineering, Amirkabir University of Technology, 424 Hafez Avenue, Tehran 59163-4311, Iran; ‡ Department of Chemistry and Materials Innovation Factory, University of Liverpool, Liverpool L69 7ZD, U.K.

## Abstract

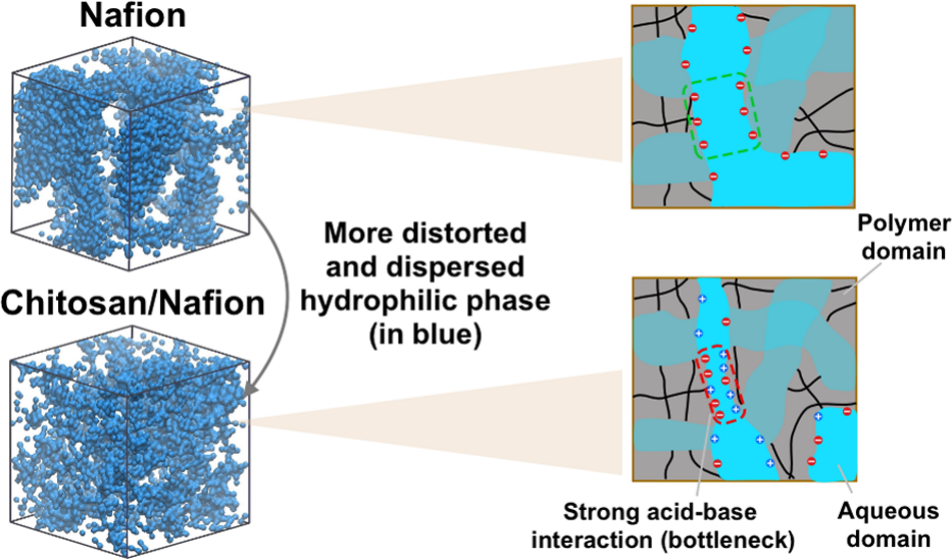

Blending a basic polymer (e.g., chitosan) with Nafion can modify some membrane properties in direct methanol fuel cell applications, e.g., controlling methanol crossover, by regulating the morphology of hydrophilic channels. Unraveling the mechanisms by which the channel morphology is modified is essential to formulate design strategies for acid–base blend membrane development. Thus, we use molecular simulations to analyze the morphological features of a blend membrane (at 75/25 chitosan/Nafion wt %), i.e., (i) water/polymer phase organizations, (ii) number and size of water clusters, and (iii) quantitative morphological measures of hydrophilic channels, and compare them to the pure Nafion in a wide range of water contents. It is found that the affinity of water to different hydrophilic groups in the blend membrane can result in more distorted and dispersed hydrophilic phase and fewer bulk water-like features compared to pure Nafion. Also, the width of the hydrophilic network bottleneck, i.e., pore limiting diameter (PLD), is found to be almost five times smaller for the blend membrane compared to Nafion at their maximum water contents. Moreover, by changing the chitosan/Nafion weight ratio from 75/25 to 0/100, we show that as Nafion content increases, all channel morphological characteristics alter monotonically except PLD. This is mainly due to the strong acid–base interactions between Nafion and chitosan, which hinder the monotonic growth of PLD. Interestingly, water and methanol diffusion coefficients are strongly correlated with PLD, suggesting that PLD can be used as a single parameter for tailoring the blending ratio for achieving the desired diffusion properties of acid–base membranes.

## 1 Introduction

Fuel cell technology is regarded as a viable option to meet the growing demand for more and cleaner energy sources; an energy conversion apparatus that converts the chemical energy of fuels to electricity with almost no environmental pollution.^1,2^ Among various types of fuel cells, direct methanol fuel cells (DMFCs) use liquid methanol as fuel and exhibit interesting characteristics, e.g., high efficiency, operating at low temperatures, using safer methanol fuel rather than explosive hydrogen fuel, and rapid refueling system.^3−5^

Proton exchange membrane (PEM) is one of the chief compartments of DMFCs which regulates the overall performance of a fuel cell by providing proton diffusion paths and impeding fuel crossover. Perfluorinated sulfonic acid (PFSA) ionomers are ion-conductive polymers that are broadly employed as PEMs. Currently, the most common PEM used in DMFCs is Dupont’s Nafion, as it affords high proton conductivity when fully hydrated along with excellent chemical and mechanical durability.^6−8^ The unique properties of Nafion stem from its hydrophobic polytetrafluoroethylene backbone and hydrophilic sulfonic acid group attached to a perfluorinated side chain. During the hydration of Nafion, the immiscibility of hydrophobic and hydrophilic segments results in a distinct phase separation which provides remarkable ion and solvent transport properties.^9^ Nevertheless, the high production cost and excessive methanol crossover of Nafion limit its application.^5^ Technically, wide interconnected diffusion pathways (water channels) caused by phase separation during hydration also trigger methanol diffusion along with water. Thus, suppressing methanol crossover can be achieved by controlling the hydrophilic channel widths.^10−12^ Proton diffusion, however, follows rather more complicated patterns. In principle, proton diffusion comprises two components, that are, vehicular and Grotthuss (or proton hopping) mechanisms. It has been reported that below the percolation threshold of water clusters, connectivity of water clusters rather than water channel widths contributes to the enhancement of proton transport, and while the percolation threshold is surpassed, increase of water channel widths contributes to further enhancement of proton transport where the Grotthuss diffusion mechanism is predominant.^13^ Therefore, any attempt to hinder methanol permeability may also reduce the proton conductivity. In terms of designing strategies, a good balance between proton conductivity and methanol permeability, i.e., membrane selectivity, is the ultimate factor determining the overall DMFC’s performance. It should be noted that methanol leakage through the membrane leads to deteriorated cell performance and unacceptable fuel efficiency; it has been reported that over 40% of the methanol can be lost across Nafion membranes.^11,14^

One practical approach to address these issues is blending Nafion with other polymers to obtain a superior PEM.^15−21^ In this line, Wycisk et al.^16^ prepared Nafion-polybenzimidazole blend membranes and studied the membrane selectivity as a function of Nafion protonation degree prior to blending. The selectivity of a blend membrane containing 8 wt % of polybenzimidazole and Nafion in its initial 100% protonation state was found to be four times greater than that of Nafion 117. The better selectivity (resulting from reduced methanol permeability) of the blend membrane was due to the acid–base interactions between imidazole and sulfonic acid groups, which hindered membrane swelling upon hydration. Somehow similar to the previous study, Zhang et al.^22^ prepared acid–base blend membranes comprised of sulfonated poly(aryl ether ether ketone) and polybenzimidazole. According to the SAXS profiles, they observed a pronounced shift of ionic scattering maximum (analogous to the ionomer peak of PFSAs, i.e., a single and broad peak corresponding to the spacing between water domains) to higher scattering vectors, which was attributed to the reduced hydrophilic channel size. The water uptake and methanol permeability of the blend membranes decreased with increasing polybenzimidazole content; the proton conductivity also decreased but not to a great extent. They concluded that the strong acid–base interactions between sulfonic acid and amine groups (hydrogen bonds) led to the compaction of the blend membranes, thereby resulting in favorable membrane properties. Their interpretation of the altered hydrated morphology of the blend membranes and its impact on methanol diffusion is applicable to the study of Wycisk et al.^16^ on Nafion-polybenzimidazole membranes. In another study, Ru et al.^23^ could reduce the methanol crossover in Nafion while enhancing the proton conductivity by introducing three kinds of sulfonated poly(arylene ether ketones) into Nafion as blending modifiers. Among them, the side-chain-type structure of the blending polymer exhibited good compatibility with Nafion. From the SAXS profiles, the ionomer peak of blend membranes was found to shift to larger scattering vectors. The selectivity of the best blend membrane was roughly four times higher than that of the recast Nafion. In that case, the enhanced selectivity was due to the decreased methanol permeability and also an increase in proton conductivity. They referred the methanol resistance to more compact membranes as a result of the compatibility of membrane components, and improved proton conductivity to the reduced activation energy (*E*_a_) of proton conduction.

In recent years, chitosan (CS), a naturally abundant and inexpensive polysaccharide, has received much attention as a promising candidate PEM mainly due to its intrinsic methanol barrier property.^24^ Chitosan is the N-deacetylated derivative of chitin; it is highly hydrophilic and insoluble in water and organic solvents. Chitosan, in its native state, demonstrates a very low ionic conductivity and a high level of swelling.^25,26^ To tackle these problems, chitosan is typically covalently or ionically cross-linked, reinforced, and blended with other polymers.^27−31^ Because of basic amino groups in the structure of chitosan, it can readily become protonated in acidic environments and become a polycation, which makes it capable of the formation of acid–base complexes with polyanions. The preparation of chitosan/Nafion blend membranes with various compositions has been reported in the literature.^32−34^ For example, Bagherzadeh et al.^34^ fabricated chitosan/Nafion blend membranes as a low-cost applicable PEM used in DMFCs. They prepared a blend membrane with the incorporation of 25 wt % Nafion into a chitosan matrix that showed a comparable selectivity to that of recast Nafion. They attributed this achievement to the reduced free volume resulting from blending Nafion with basic chitosan. It led to the diminished methanol permeability and only a marginal decrease in proton conductivity due to the reduced activation energy of proton conduction (facilitating proton diffusion).

Diffusion in PEMs, in essence, depends on the hydrated morphology. In other words, the geometry of hydrophilic nanochannels is an important membrane characteristic that informs the quality of diffusants (ion, water, and methanol) transportation inside the membrane.^35−38^ By scattering studies, one can determine the average spacing of hydrophilic domains but their shape, structure, or connectivity cannot be directly elucidated. Furthermore, the structure-transport relationships often remain unknown due to the inhomogeneous nature of polymeric materials.^9,39^ Simulation techniques, as complementary research tools to experiments, have significant capability to unravel molecular understanding of such complex mediums.^40^ Hydrated membrane morphology, structural features, and morphology-transport interrelation in PEMs (particularly for PFSAs) have been extensively studied by atomistic molecular dynamics (MD) simulations^41−50^ and mesoscale simulations such as coarse-grained (CG) MD^51,52^ and dissipative particle dynamics.^53−56^ However, systematic morphological study on blend PEMs is quite scarce in the literature, and their nanoscale features are still important to understand.

In the current study, we constructed CG models of Nafion and chitosan/Nafion blend membranes. After performing several layers of verification simulations for our models, we ran a series of MD simulations on hydrated membranes to investigate the hydrophilic morphology of a blend membrane (at a specific blend ratio) and compare it to that of a pristine Nafion membrane through (i) quantification of membrane morphology, (ii) water cluster analysis, and (iii) calculation of geometrical parameters for hydrophilic pathways at different water contents. Then, we analyzed the hydrophilic morphology of blend membranes with varying chitosan/Nafion ratios at a constant water content (their maximum water content). Also, we explored the correlation between water network morphological parameters and the diffusion rate of water/methanol to formulate their relationships. Note that based on what was stated previously, i.e., the major role of Grotthuss mechanism in proton diffusion at high water contents, and the inability of our CGMD simulations to capture this mechanism, our study is not framed for systematic investigation of relationships between hydrated morphologies and proton diffusion.

## 2 Methods

### 2.1 Materials

**Figure 1:**
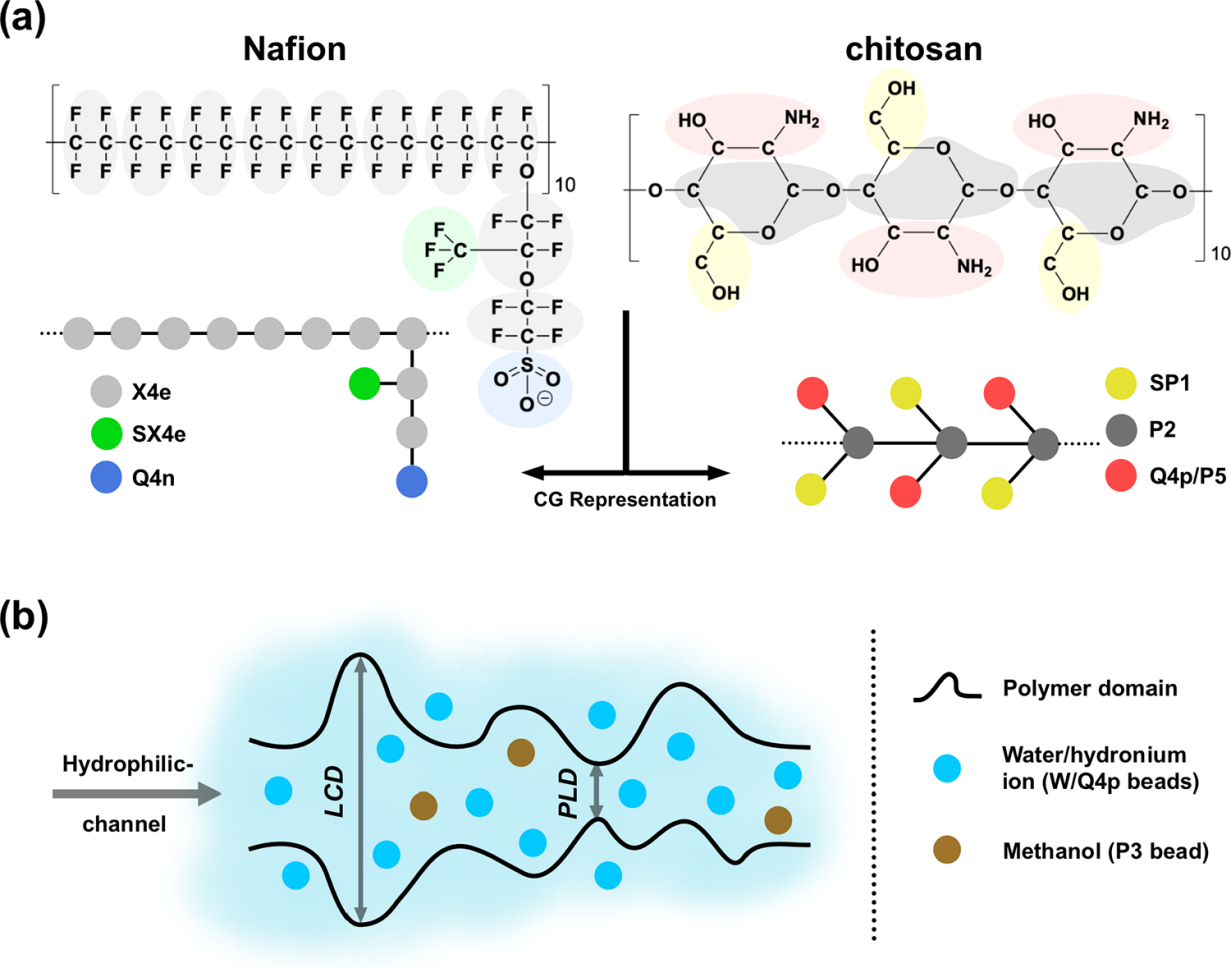
(a) Molecular structure and subsequent CG representation of Nafion and chitosan along with bead types based on Martini 3 force field, (b) hydrated membrane structure and schematic definition of pore limiting diameter (PLD) and largest cavity diameter (LCD) characteristics.

The Nafion model has an equivalent weight (*EW*) of 1144 g/mol (representing its common commercial structure). Each Nafion chain consists of 10 monomers terminated by SO_3_H groups (*M*_*n*_ = 11580 g/mol) (Figure 1a). For chitosan, the model has 30 monomers, and it is in the 100% deacetylated state (*M*_*n*_ = 4852 g/mol) (Figure 1a). The mentioned length and structure of Nafion and chitosan chains have been previously adopted by researchers in their MD simulation studies.^42,43,57,58^

### 2.2 Molecular Modeling

We employed the Martini 3 model for coarse-graining our membrane systems.^59^ The CG structures and bead types for both polymers are shown in Figure 1a. The bonded interactions between beads for Nafion were calculated by mapping from all-atom (AA) MD simulations. CG structuring and bead interactions for chitosan were adopted from Xu et al. study.^57^ More details about AA simulations and CG parametrizations are provided in the Supporting Information (SI), Sections 1 and 2.

After verification of our CG model (discussed later in Section 3), we created the hydrated blend membranes by deprotonating SO_3_H groups of Nafion to SO_3_^–^ via changing its bead typing from P3d to Q4n, and incorporation of sulfuric acid (H_2_SO_4_) as chitosan cross-linker in its ionized state (SO_4_^2–^, sulfate ion), and finally, assigning every H^+^ derived from Nafion and sulfuric acid to chitosan ionizable amine groups (no free ions exist) randomly by switching its bead typing (from P5) to Q4p (see Figure 1a). This concept is consistent with the (experimental) work of Bagherzadeh et al.,^34^ in which they obtained cross-linked chitosan/Nafion blend membranes.

For convenience, we named chitosan/Nafion blend membranes at *xx*/*yy* ratio as blend-*yy*, where *yy* refers to Nafion wt % in a blend membrane (*xx* = 100 – *yy*). For instance, blend-25 refers to chitosan/Nafion at a 75/25 ratio. We sampled one of the blend membranes, i.e., blend-25, at five different water contents (WC, defined as the ratio of water mass to dry membrane mass), 50, 40, 30, 20, and 10%. The maximum water content of 50% corresponds to the experimental water uptake of blend-25 membrane.^34^ We also sampled a pure Nafion membrane at seven different WCs, 35, 30, 25, 20, 15, 10, and 5%. In our case, WC of 35% corresponds to the hydration level (λ, number of water molecules per sulfonate group) of 22.5 which is close to the experimental maximum liquid water uptake of Nafion 117.^60^ Note that Nafion is fully deprotonated at λ ≥ 3,^61^ which is below the lowest WC that we considered in our study; therefore, Nafion is considered fully deprotonated at all WCs and 1000 Q4p-type beads were assigned to hydronium ions existing in hydrated Nafion systems.

We constructed blend-50 and blend-75 membranes at their maximum WCs. Their maximum WCs were determined by interpolation of blend membranes and Nafion maximum WCs data and their sulfuric acid contents by extrapolation of blend membranes sulfuric acid data found in ref (34).^34^ Separate simulations with additional 1000 methanol beads and 1000 hydronium ions (with same amount of negatively charged beads to preserve system neutrality) for blend systems and 1000 methanol beads for the Nafion system (hydronium ions already exist) were performed to calculate CG diffusion coefficients for section 3–3 of the article. Tables 1 and 2 and Table S2 show the full details of simulated systems and bead typing with details, respectively.

**Table 1:** Composition of Nafion and Blend-25 Membranes at Different Water Contents (WCs)

	no. chitosan chains	no. Nafion chains	no. SO_4_^2–^ ions	no. water molecules	no. water beads	WC (wt %)	λ
Nafion	0	100	0	22,516	5379	35%	22.5
	0	100	0	19,300	4575	30%	19.3
	0	100	0	16,083	3770	25%	16.08
	0	100	0	12,866	2966	20%	12.86
	0	100	0	9650	2162	15%	9.65
	0	100	0	6433	1358	10%	6.43
	0	100	0	3216	554	5%	3.21
blend-25	105	15	870	21,344	5336	50%	
	105	15	870	17,076	4269	40%	
	105	15	870	12,804	3201	30%	
	105	15	870	8536	2134	20%	
	105	15	870	4268	1064	10%	

**Table 2:** Composition of Nafion and Blend Membranes with Varying Chitosan/Nafion Ratios at Their Maximum Water Content (WC)

	no. chitosan chains	no. Nafion chains	no. SO_4_^2–^ions	no. water beads	WC (wt %)
blend-25	105	15	870	5336	50%
blend-50	85	34	680	5333	44%
blend-75	48	60	420	5113	38%
Nafion	0	100	0	5379	35%

### 2.3 Simulation Details

We employed GROMACS (version 2020–2)^62^ for all MD simulations. An identical equilibration procedure was performed for all of the membrane systems. Components of the membranes (according to Tables 1 and 2) were randomly inserted in a 30 nm × 30 nm × 30 nm simulation box. After energy minimization, equilibration took place in six consequent steps and was followed by a production run: (1) a NVT run of 3 ns at *T* = 300 K, (2) a NPT run of 2 ns at *T* = 300 K and *P* = 100 bar, (3) a NPT run of 2 ns at *T* = 300 K and *P* = 1 bar, (4) a NVT run of 2 ns at *T* = 500 K followed by a NPT run of 5 ns at *P* = 1 bar and the same temperature, (5) a NVT run of 2 ns at *T* = 400 K followed by a NPT run of 5 ns at *P* = 1 bar and the same temperature, (6) a NVT run of 5 ns at *T* = 300 K followed by a NPT run of 60 ns at *P* = 1 bar and the same temperature, and finally, a NPT production run of 5 ns at *T* = 300 K and *P* = 1 bar for data collection. The first and last 1 ns of production runs were ignored for all analyses. The leapfrog integration algorithm was used for simulations and periodic boundary conditions in three dimensions were applied. The temperature and pressure of the simulations were controlled by a V-rescale thermostat and a Parrinello–Rahman barostat, respectively. A time step of 5 fs for NVT and 10 fs for NPT simulations was set. The VMD software^63^ was used for the visual illustration of the membrane systems.

### 2.4 Analyses

We utilized the radial distribution function (RDF) g_A-B_ (r) to characterize the bead–bead interactions in the CG membrane systems. RDF can be defined as the probability of local distribution of particle A around particle B as a function of distance.^64^ We also used the coordination number (CN), i.e., the average number of particles in a given distance of a reference particle,^37^ to quantify the bead–bead interactions and elucidate the occurrence of cross-linking in blend membranes.

Water cluster analysis was applied to study the evolution of interconnected water networks within membranes upon hydration. In our study, a water cluster in a membrane was considered a group of water beads (and hydronium beads if exist), in which each water bead is within a predetermined cutoff distance of at least another water bead in that group. The cutoff distance was set to 0.724 nm corresponding to the first minimum of water–water RDF in Nafion and blend membranes at 300 K.

To probe the hydrophilic morphology of the membranes, we employed Poreblazer (V4.0) software.^65^ It can compute some quantitative characteristics of the channel structure in membranes including the largest cavity diameter (LCD), pore limiting diameter (PLD), network-accessible surface area (*S*), and pore-occupiable volume (*V*). A schematic definition of LCD and PLD is shown in Figure 1b. For this purpose, all water beads (and hydronium beads if exist) were excluded from the equilibrated simulation box to achieve a porous-like polymeric matrix as an input for Poreblazer calculations. The results are reported by averaging the data over four different frames of production runs.

The Einstein formula was used to determine the diffusion coefficients for water beads (*D*_w_), methanol beads (*D*_m_), and hydronium ions (*D*_h_):
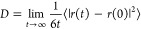


In the above formula, *r*(*t*) is the center of mass position vector of mentioned beads at time *t*.^43^

## 3 Results and Discussion

### 3.1 Verification of Simulated Systems

To evaluate whether the simulated systems were equilibrated in the last 60 ns of the equilibration procedure (mentioned in Section 2–3), we used the time autocorrelation function of the radius of gyration of polymeric chains (see the SI, Section 3–1). As shown in Figure S3, the squared radius of the gyration autocorrelation function dropped to zero in early times for polymeric chains in Nafion and blend-25 systems at their minimum water contents. This indicates the relaxation of polymeric chains, which have the slowest relaxations in the systems. Therefore, 60 ns is long enough for the equilibration of the systems. We also performed some system size analyses to check the independency of our results from the box sizes (see the SI, Section 3–2). According to Figures S4 and S5, the effect of the system size on our results is negligible.

To verify our CG simulations, for the Nafion membrane system, we compared the equilibrated CG densities with experimental values. The densities of the hydrated CG Nafion system showed up to 4% drift from the experimental data^66^ (see the SI, Section 3–3). We also qualitatively compared our CG water and hydronium ion diffusion coefficients with relevant atomistic MD simulation studies.^43,67^ It has been found by experiments and atomistic MD simulations that water and hydronium diffusivities increase with increasing water content due to the reduction of confinement. The water–water coordination number has been proposed as a measure of confinement in atomistic MD simulations.^68^ Within our CG results, we also observed a reduction in the CG water–water coordination number (will be discussed later in Section 3–2–1) and an increase in the water diffusion coefficient (accompanied by the increase of the hydronium ion diffusion coefficient) with increasing water content (see the SI, Section 3–3). However, CG diffusivities must not be compared quantitatively by either experiments or atomistic simulations, due to the smoothness of the CG potential energy landscape,^69^ and any quantitative match must be accidental. Just insightful qualitative trends can be compared.^69^ Verification of our CG Nafion models is not limited to comparing the densities and diffusion trends, but multiple other aspects of the models, e.g., RDFs, water clustering, aqueous surface to total box volume ratio, etc., which will be mentioned throughout this article, justify the correctness of our calculations. Lastly, the CG density of the blend-25 system was found to be 1350 kg/m^3^ (at WC = 50%) which is close to its experimental value^34^ (1301 kg/m^3^). Therefore, due to the implementation of a verified CG Martini model of chitosan and Nafion, we are confident that our CG simulations are valid for further analysis and interpretation in the rest of this study.

### 3.2 Role of Hydration on Morphological Characteristics

#### 3.2.1 RDF and Coordination Number

**Figure 2:**
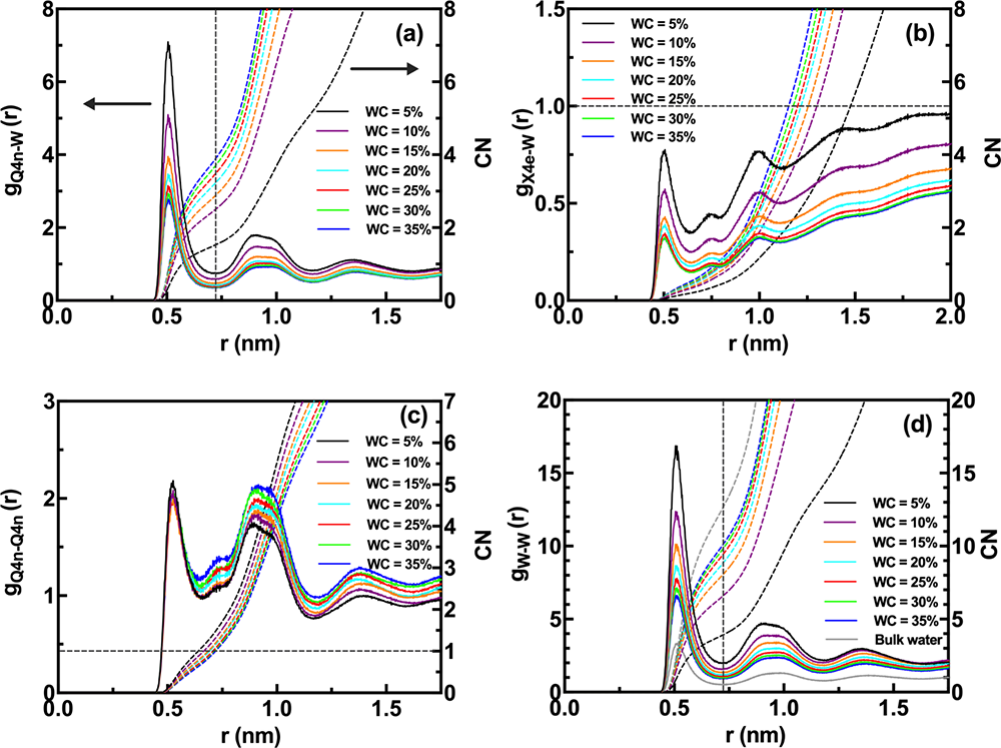
RDF (solid lines, left *y*-axis) and CN (dashed lines, right *y*-axis) of (a) Nafion sulfonate-water beads, Q4n-W, (b) Nafion fluorinated-water beads, X4e-W, (c) Nafion sulfonate-sulfonate beads, Q4n–Q4n, and (d) water–water beads, W–W, for the Nafion system as a function of water content (WC).

The affinity of water toward different polymer groups can regulate the water channel morphology in PEMs. Figure 2a displays Nafion sulfonate-water beads (Q4n-W) RDFs for the Nafion system. The decreasing height of the maximum peaks of RDFs with increasing WC is similar to previous studies.^39,42,43,70^ Based on CN graphs, the average number of water beads around Nafion sulfonate beads increases as WC increases. This is due to the greater solvation of hydrophilic sulfonate groups by water upon hydration. Nafion fluorinated-water beads (X4e-W) RDFs are displayed in Figure 2b. The average local number density of water beads within 2 nm of the backbone fluorinated beads remains lower than the average number density of water beads in the simulation box (i.e., *g*(*r*) < 1), which is due to the hydrophobic nature of the Nafion backbone. Figure 2a,b obviously demonstrate a distinct phase separation in the Nafion system upon hydration, as expected. Also, it has been previously shown that Nafion sulfonate groups become more separated with increasing membrane hydration.^42,43^ The same observation can be concluded from the sulfonate-sulfonate beads (Q4n–Q4n) RDFs, as shown in Figure 2c. According to the CN graphs, the average distance from a sulfonate bead within which another sulfonate bead can be found (CN = 1) increases with increasing WC (intersection of the horizontal line with CN graphs in Figure 2c). Lastly, Figure 2d shows the structure of water beads in Nafion. As shown, by increasing WC, the W–W RDFs resemble more bulk water-like RDF, i.e., increasing W–W coordination numbers upon hydration. This implies that with increasing WC, the local confinement decreases, which is a common result from atomistic simulations.^42,68,70^

**Figure 3:**
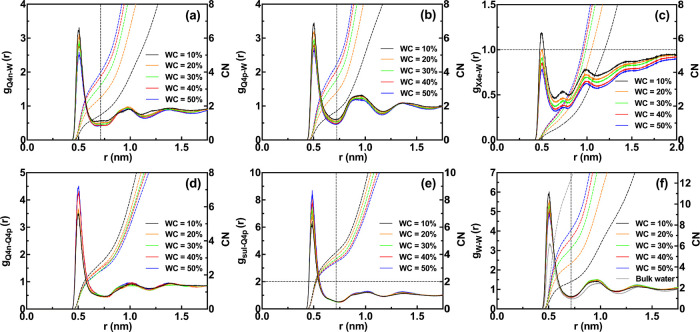
RDF (solid lines, left *y*-axis) and CN (dashed lines, right *y*-axis) of (a) Nafion sulfonate-water beads, Q4n-W, (b) chitosan amine-water beads, Q4p-W, (c) Nafion fluorinated-water beads, X4e-W, (d) Nafion sulfonate-chitosan amine beads, Q4n–Q4p, (e) sulfate ion-chitosan amine beads, sul-Q4p, and (f) water–water beads, W–W, for the blend-25 system as a function of water content (WC).

Similar RDF analyses for the blend-25 system were performed, and the results are shown in Figure 3. Figure 3a,b show Nafion sulfonate-water (Q4n-W) and chitosan amine-water beads (Q4p-W) RDFs for the blend-25 system, respectively. In both figures, because of the solvation of these groups with water beads, CN tends to increase upon hydration. It is worth mentioning that the maximum CN value corresponding to Q4n-W RDF for the blend-25 system (intersection of the vertical line with CN graph at WC = 50% in Figure 3a) is 3.74, which is less than the maximum CN value for the Nafion system (intersection of the vertical line with CN graph at WC = 35% in Figure 2a), i.e., 3.9. This is due to the presence of other hydrophilic groups in the blend-25 system, i.e., all three chitosan beads (in each chitosan repeating unit) and sulfate ions, being able to attract water beads, which may lead to distorted water channel morphology compared to pure Nafion membrane. The less pronounced phase separation can be further concluded from Nafion fluorinated-water bead (X4e-W) RDFs, as shown in Figure 3c. The intensity of RDFs at all WCs is higher than that of the Nafion system, and at some WCs (i.e., WC = 10% and WC = 20%), RDFs even surpass the horizontal line (g(r) > 1). The Nafion sulfonate-chitosan amine beads (Q4n-Q4p) RDFs are illustrated in Figure 3d. Because of the strong acid–base interaction between these groups, the RDFs show a sharp peak at around 0.5 nm. Upon an increase in the water content, the average interaction between these groups decreases, which is most likely due to the better solvation of both groups by water. As stated previously, sulfate ions do exist in the blend-25 system as the chitosan cross-linking agent. Figure 3e shows sulfate ion-chitosan amine bead RDFs and CN values. At all WCs, CN values are above 2 (intersection of the vertical line with CN graphs), which is a proof of cross-linking in the system (each sulfate ion is surrounded with more than two chitosan amine beads). Lastly, the water–water beads (W–W) RDFs of the blend-25 system (Figure 3f) show a decreasing trend in their maximum peak height just like the Nafion system, approaching the bulk water RDF at WC = 50%. Nevertheless, the maximum CN value in this system (intersection of the vertical line with CN graph at WC = 50% in Figure 3f), i.e., 7.67, is less than that of the Nafion system, i.e., 10.14. This shows that water in the Nafion system, at its maximum WC, exhibits more bulk water features and is less confined.

#### 3.2.2 Water Cluster and Hydrophilic Morphology

A well-interconnected hydrophilic network is vital for obtaining high-performance PEMs.^37^ For this purpose, we analyzed the number of water clusters and normalized cluster size as a function of WC for both the Nafion and blend-25 systems. The normalized cluster size is defined as the ratio of the largest cluster size to the whole number of water beads (and hydronium beads if exist) in the simulation box. This quantity gives us insight into the relative size of the interconnected water network and the hydration percolation threshold of the membranes.

**Figure 4:**
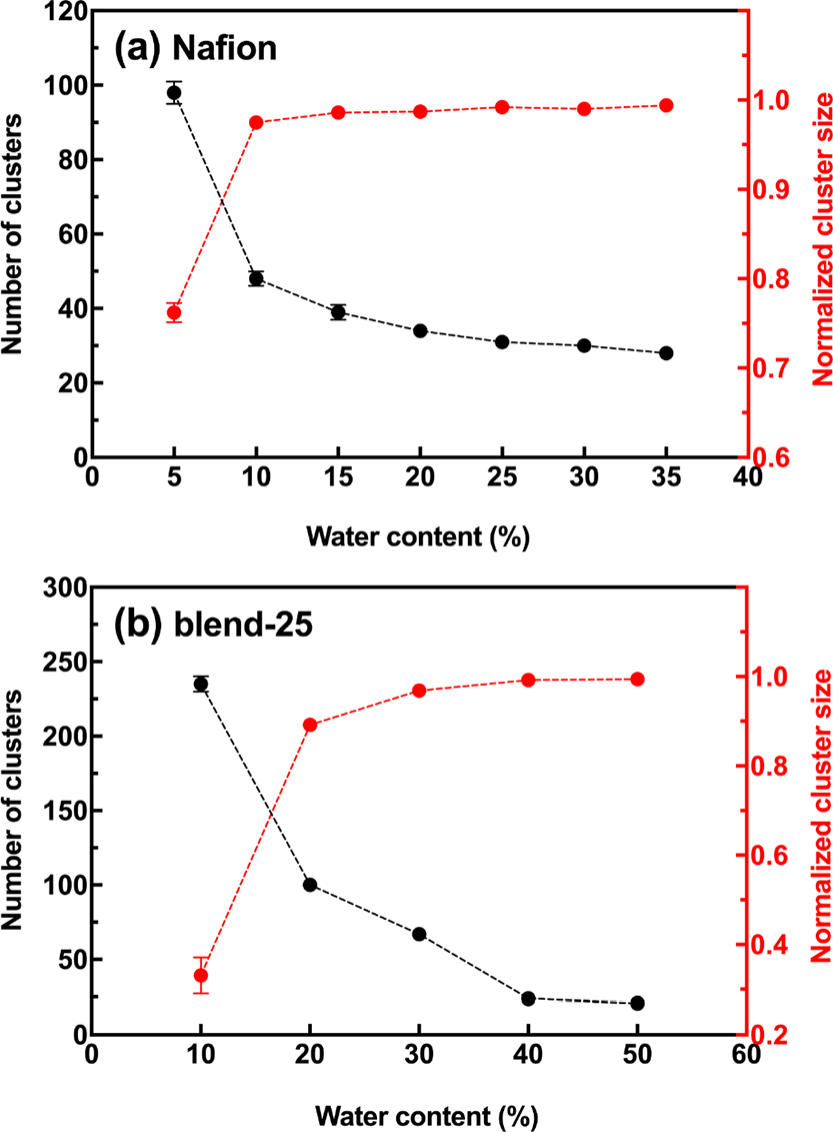
Number of water clusters (in black) and normalized cluster size (in red) as a function of water content for (a) Nafion system and (b) blend-25 system.

Figure 4 demonstrates the number of water clusters and normalized cluster size as a function of the WC for both membrane systems. From Figure 4a for the Nafion system, water clusters are highly isolated and large in number at WC = 5% and they tend to decrease in number with increasing WC (merging water clusters upon more hydration). Also, at WC = 5%, 76% of water beads and hydronium ions belong to the largest cluster and this number increases to near 98% at WC = 10%. This means that there is a single percolated water channel at WC = 10% and beyond. Thus, our models predict a percolation threshold between WC = 5% and WC = 10% for Nafion, which is in agreement with the prediction of Devanathan et al.^71^ of occurring water network percolation between λ = 5 and λ = 6 (equivalently, WC = 7.7% and WC = 9.3%). For the blend-25 system (Figure 4b) also, the merging of isolated water clusters with increasing WC can be seen. Nonetheless, the number of water clusters at the minimum WC (WC = 10%) is almost 2.5 times bigger than that of the Nafion system. Furthermore, the change in cluster size as a function of WC is more gradual as compared to the Nafion system. This suggests a different hydrophilic morphological change upon membrane hydration for Nafion and blend-25 systems. At maximum WCs, i.e., WC = 50% for blend-25 and WC = 35% for Nafion, however, both membranes show a completely interconnected water channel throughout the simulation boxes.

**Figure 5:**
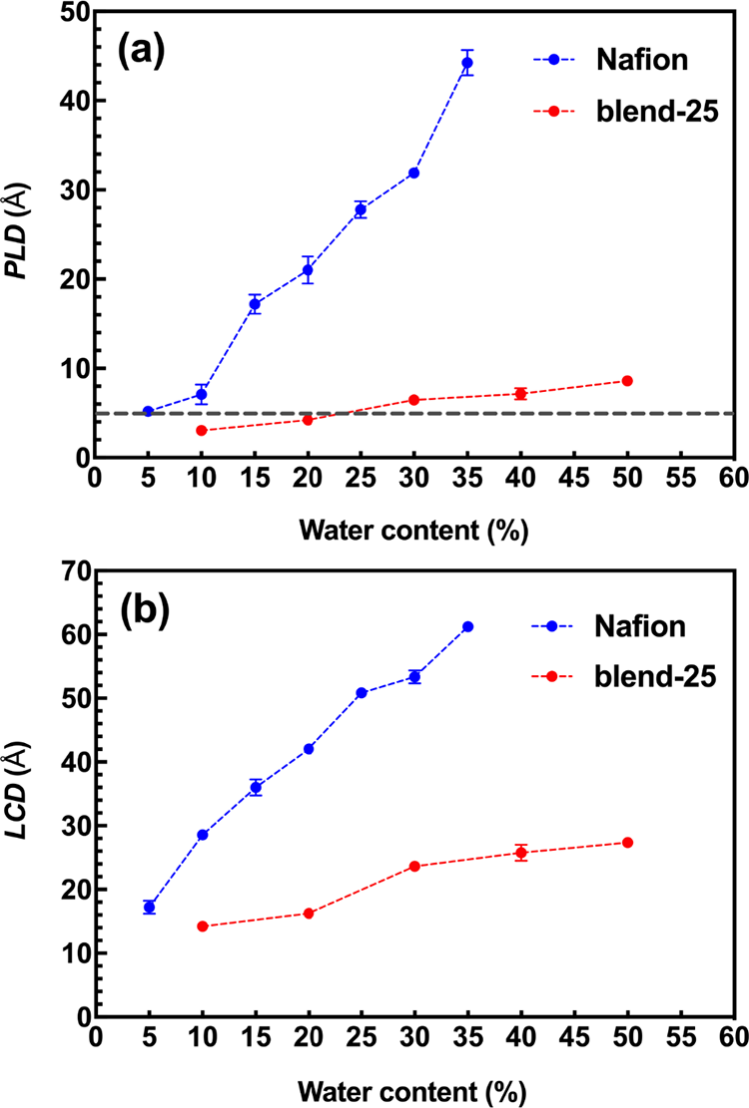
(a) Pore limiting diameter (PLD) and (b) largest cavity diameter (LCD) as a function of water content for Nafion and blend-25 systems.

In PEMs, water molecules are confined in the pores of heterogeneous polymer structure, and diffusion occurs within these filled water pores that are connected by bottlenecks. Therefore, the size of bottlenecks and pores are two restricting geometrical factors that determine the diffusion quality of any diffusant, e.g., water, hydronium ion, and methanol. Quantifying bottlenecks and pores diameter can be possible by calculating two morphological measures of the membranes, i.e., PLD and LCD, respectively, which were previously defined (Figure 1b). PLD could be considered as a critical parameter for diffusion; molecules (in our case, beads) can only be transported from one pore to an adjacent pore only if the bottleneck size connecting the two pores surpasses a threshold value.^37,64,72^ In our study, the threshold value is considered the diameter of a water bead, i.e., 4.7 Å (illustrated with a dashed line in Figure 5a), according to Martini 3 model.^59^

In Figure 5a, the increase of PLD upon hydration is evident for both membranes. For Nafion and blend-25, at WC = 5% and WC = 20%, PLD falls on the threshold value, respectively. These are completely in line with the normalized cluster size results that were mentioned earlier. To be more specific, as PLD exceeds the threshold value, the complete interconnection of water clusters results in a spanning water network in the hydrated membrane. The fully interconnected water network plays a dominant role in diffusion. Hence, PLD is an essential parameter in assessing both hydrated morphology and diffusion. Almost at all WCs, the PLD of Nafion is considerably greater than that of blend-25, and at the maximum WC of both systems, the PLD of Nafion is almost five times larger than that of blend-25. It is also worth mentioning that the slope of the PLD increase in Nafion is clearly larger than in blend-25, meaning that the hydrated morphology of the Nafion system is more sensitive to hydration than the blend-25. LCD value gives the largest pore diameter in a hydrated membrane. As displayed in Figure 5b, similar to PLD, LCD rises as WC increases in both membrane systems, but all LCD values of blend-25 are smaller than those of Nafion. Also, our models predict PLD = 27.78 Å and LCD = 50.86 Å for Nafion at WC = 25% (λ = 16.08), which are close to the reported 3–5 nm diameter of water domains based on the SAXS experiment.^70^

Here, we gained a holistic view of hydrated morphology in both membrane systems through some quantitative parameters along with their differences and similarities. We further wish to have a geometrical view of the hydrated morphologies as well. We used the surface-to-volume ratio (*S*/*V*) of the aqueous domain to compare the morphology of the hydrophilic phase. The S/V is a suitable quantitative parameter to describe the size and geometry of an object. A rise in the S/V means that (1) the size of the object decreases or (2) the shape of the object becomes distorted and elongated (less spherical). Furthermore, the ratio of the aqueous domain surface area (*S*) to the total volume of the membrane (TV) can be utilized to explain the distribution of water in the hydrated membrane. A larger *S*/TV ratio signifies the dispersion of water in the membrane rather than the aggregation in clusters.^43^

For having accurate comparison and excluding the object size effect at this point, Nafion and blend-25 systems at water contents with the same amount of water molecules (Table 1) should be compared. To this end, Nafion at WC = 35% and blend-25 at WC = 50%, and Nafion at WC = 20% and blend-25 at WC = 30% can be compared side by side, respectively. The *S*, *V*, TV, *S*/*V*, and *S*/TV values of Nafion and blend-25 systems are listed in Table 3. Both *S*/*V* and *S*/TV ratios of the blend-25 system at WC = 50 and 30% were found to be higher than those of the Nafion system at WC = 35 and 20%. It reveals that the hydrophilic phase is more distorted in shape (less spherical) and also more dispersed throughout the blend-25 system compared to the Nafion system. This result is in line with the smaller W–W coordination numbers of the blend-25 system. As mentioned earlier, this phenomenon can be ascribed to the presence of several distinct hydrophilic groups in the blend-25 membrane, capable of attracting water beads separately and leading to more distorted water channels and less pronounced phase separation compared to the hydrated Nafion membrane.

**Table 3:** Aqueous Surface Area (*S*) and Volume (*V*), and Total Volume (TV) of Nafion and Blend-25 Systems at Different Water Contents (WCs)

	WC (wt %)	aqueous surface area (nm^2^)	aqueous volume (nm^3^)	total volume (nm^3^)	*S*/*V* (1/nm)	*S*/TV (1/nm)	*S*/TV (1/nm)(Exp)^43,73^
nafion	35%	897.491	777.445	1540.154	1.154	0.582	
	30%	886.061	685.440	1443.199	1.292	0.613	0.38_@ λ=20_
	25%	816.821	579.219	1347.945	1.410	0.605	0.51_@ λ=15_
	20%	856.798	506.933	1269.696	1.690	0.674	0.52_@ λ=12_
	15%	784.174	393.351	1155.785	1.993	0.678	0.60_@ λ=9_
	10%	670.650	283.902	1063.203	2.362	0.630	0.53_@ λ=6_
	5%	217.192	90.401	971.332	2.402	0.223	0.47_@ λ=3_
blend-25	50%	1541.676	665.66	1378.747	2.316	1.118	
	40%	1238.561	537.380	1259.929	2.304	0.983	
	30%	827.592	367.419	1144.149	2.252	0.723	
	20%	471.351	214.396	1028.876	2.198	0.458	
	10%			952.434			

In the blend-25 system, both *S*/*V* and *S*/TV ratios increase with increasing WC, indicating that the hydrophilic phase becomes more distorted and dispersed upon hydration. This result is logical since the different hydrophilic groups can pull away water beads more noticeably. On the contrary, the *S*/*V* ratio in the Nafion system decreases with increasing WC; this is also reasonable because of more phase-separated morphology and more aggregated water domains with larger sizes. The general trend of our *S*/TV values for the Nafion system is in notable agreement with experimental data (last column in Table 3), which shows the precision of our CG Nafion model to reproduce the morphology of the hydrated Nafion membrane.

**Figure 6:**
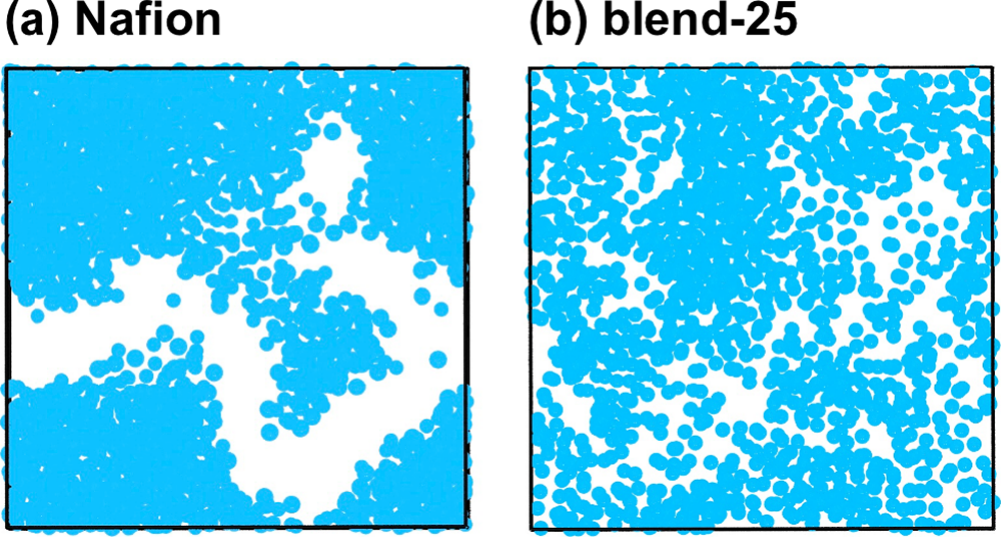
2D snapshots of (a) Nafion at its maximum water content (WC = 35%) and (b) blend-25 at its maximum water content (WC = 50%). Water beads (and hydronium beads if exist) are colored in blue, and polymer chains are excluded from the snapshots (white areas).

To have a better visualization of different water network morphologies of Nafion and blend-25 systems, two snapshots of these systems at their maximum water contents (Nafion at WC = 35% and blend-25 at WC = 50%) are displayed in Figure 6. As seen, water clusters are perfectly aggregated and form an interconnected water network in Nafion (Figure 6a). On the other hand, for blend-25, water clusters are highly dispersed, while the interconnected water network is still formed (Figure 6b). Further snapshots of both systems to see the evolution of their water network morphology upon hydration can be found in Figure S8.

### 3.3 Role of the Nafion Content on the Hydrophilic Morphology and Diffusion

**Figure 7:**
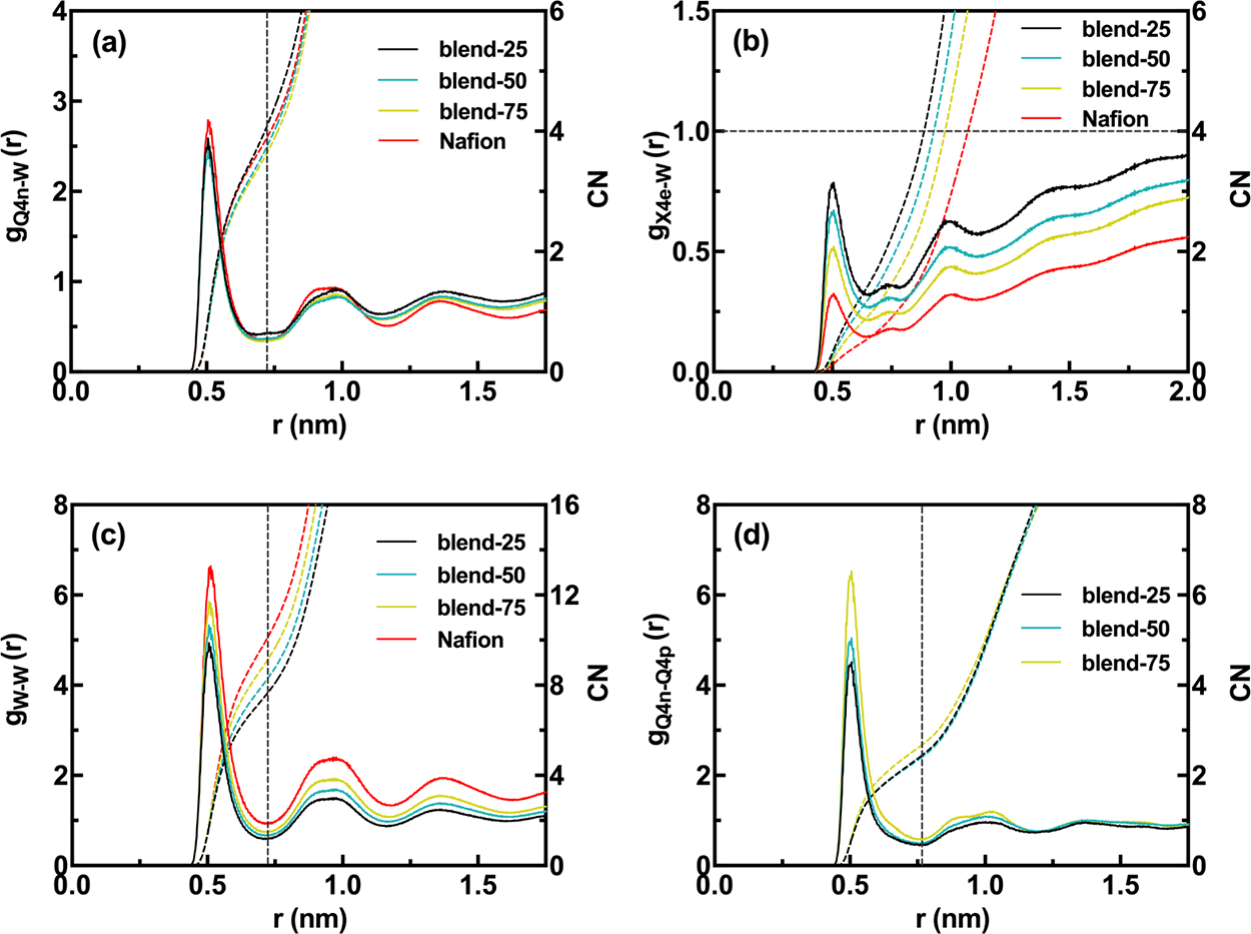
RDF (solid lines, left *y*-axis) and CN (dashed lines, right *y*-axis) of (a) Nafion sulfonate-water beads, Q4n-W, (b) Nafion fluorinated-water beads, X4e-W, (c) water–water beads, W–W; and (d) Nafion sulfonate-chitosan amine beads, Q4n–Q4p, for blend systems as a function of Nafion content.

In this section, we evaluate how the Nafion content affects the morphological characteristics and diffusion of water, methanol, and hydronium. In Figure 7, the corresponding RDFs of blend systems as a function of Nafion content are displayed. Figure 7a shows the Nafion sulfonate-water beads (Q4n-W) RDFs. Apparently, the effect of the Nafion concentration on this interaction is negligible. Figure 7b shows the Nafion fluorinated-water beads (X4e-W) RDFs. As expected, one can see that the hydrophobicity increases as the Nafion content increases. Figure 7c also illustrates the water–water beads (W–W) RDFs. The maximum intensity of RDFs and CN values increase and reach to the corresponding value for the pure Nafion. This shows that water exhibits more bulk water features as Nafion content increases. The existence of an acid–base interaction between Nafion sulfonate and chitosan amine beads can be inferred from Figure 7d at all Nafion contents due to their sharp peaks at around 0.5 nm. This suggests that with increasing Nafion content, phase separation becomes more pronounced, but strong electrostatic interactions are still present in blend systems which may lead to unusual diffusion behavior. For more clarity and visual insight into the Nafion content effect on hydrophilic morphologies, snapshots of blend membranes are presented in Figure S10.

**Figure 8:**
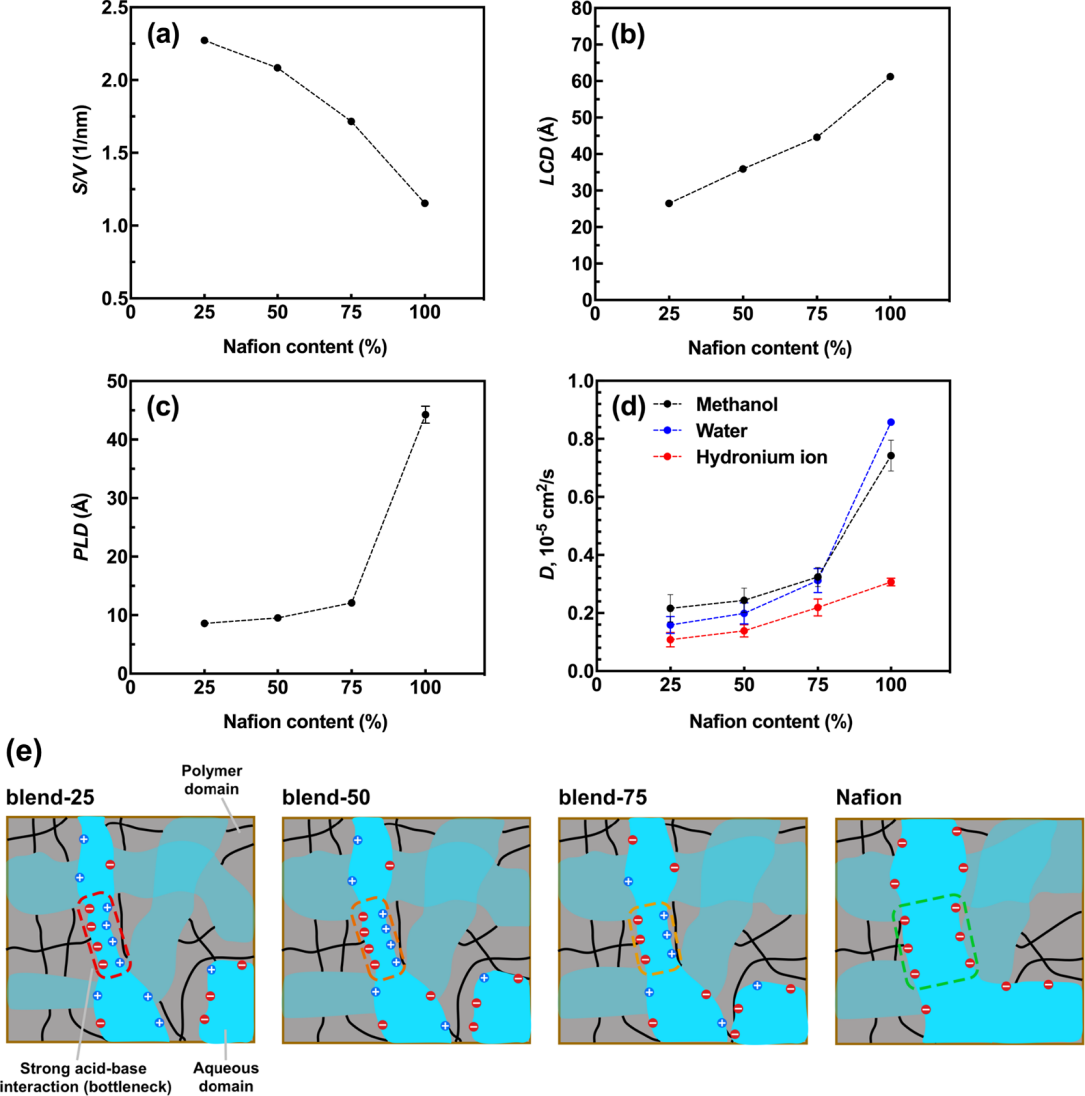
(a) Surface-to-volume ratio (*S*/*V*) of the aqueous domain, (b) largest cavity diameter (LCD), (c) pore limiting diameter (PLD), and (d) diffusion coefficients for blend systems as a function of the Nafion content. (e) Schematic of hydrophilic phase transition upon Nafion increment in blend systems.

As displayed in Figure 8a,b, *S*/*V* decreases and LCD increases monotonically with the increasing Nafion content from 25 to 100%. This means that the hydrophilic phase increases in diameter and tends to be less distorted (more spherical) as Nafion content increases in blend systems. In Figure 8c, as expected, PLD also grows as the Nafion content increases in blend systems. Between blend-75 and Nafion systems, a jump in PLD value can be observed, and prior to this jump, the increase of PLD is rather smooth. As discussed before, PLD is expected to be correlated with the diffusion coefficient. Similar to Figure 8c, an obvious jump in water and methanol diffusion coefficients can also be seen in Figure 8d, but the same is not true for the hydronium ion diffusion coefficient. This is because of the more complicated diffusion mechanism of protons in PEMs; in our study, we just treated protons as hard spheres (hydronium ions) and we only accounted for vehicular diffusion. Based on our findings from Figures 7 and 8, one can conclude that the addition of Nafion strengthens phase separation in blend systems, but it cannot change the width of hydrophilic channel bottlenecks noticeably and water/methanol diffusion rates subsequently due to the presence of strong acid–base interactions. Figure 8e schematically exhibits the transition of hydrophilic phase and bottlenecks upon Nafion increment in the blend systems. Note that this scheme is presented for better understanding of Figure 8a,b, and especially Figure 8c; the scheme is conceptual, and it should not be comprehended as genuine in terms of even charge distribution on either side of the bottlenecks. 2D snapshots showing charge distribution in these systems are also provided in Figure S10. Moreover, to better illustrate the correlation between the water diffusion coefficient and PLD, a universal plot of the water diffusion coefficient against PLD of all model membranes is shown in Figure S11.

Previously, Rezayani et al.^37,64,72^ established the correlation between the PLD parameter and water/methanol diffusivities in several hydrated ionomer systems. Here, we further showed this correlation in an acid–base model membrane and explained by which way the PLD parameter is controlled, i.e., strong acid–base interactions.

## 4 Conclusions

The main drawback of Nafion membranes is a considerable methanol crossover due to the wide interconnected water channels. To address this issue, blending Nafion with basic polymers was found to be a viable solution. In this line, we studied chitosan/Nafion blend membranes, in which it is expected that chitosan mediates the phase separation between membrane and water and controls the width of water channels. Our molecular simulations show that blend membranes adopt a more distorted and dispersed hydrophilic phase compared to the pure Nafion membrane. Consequently, the width of hydrophilic network bottlenecks in the blends, i.e., PLD, is much less than that of Nafion. This is mainly due to the existence of more hydrophilic groups and strong acid–base interactions in blend membranes that can hinder the extent of phase separation. We also observed a clear correlation between PLD and the water/methanol diffusion coefficient in blend membranes as a function of Nafion content. Based on our findings, a minor addition of a basic polymer (in our case, chitosan) to Nafion can significantly alter the hydrophilic morphology, hampering the unfavorable methanol diffusion by reducing the width of the water network bottleneck. Thus, we conclude that the inclusion of a minor portion of a basic polymer to an excellent acidic proton-conducting polymer, e.g., PFSAs or other sulfonated PEMs, can substantially reduce the methanol crossover so that the selectivity of the membrane is preserved regardless of the decrease in proton conductivity. This study also provides a general framework for in silico testing of similar blend membranes in terms of quantification of membrane hydrophilic morphology by introducing PLD as a single coefficient strongly correlating with the water/methanol (and any other solvent) diffusive properties.

## Supporting Information Available

Atomistic simulation details; coarse-grained (CG) parameters; validation of models; whole box RDFs; snapshots of hydrated membranes; MSD plots; snapshots of blend membranes at their maximum water content; and universal plot of water diffusion coefficients versus PLD (PDF)
