# Supplementary material for: Morphology and Transport Study of Acid–Base Blend Proton Exchange Membranes by Molecular Simulations: Case of Chitosan/Nafion
Source: J Phys Chem B. 2023 Dec 1;127(49):10624–35. doi: 10.1021/acs.jpcb.3c05332 (PMC10726362; doi:10.1021/acs.jpcb.3c05332)
Supplement: Supplementary file 1 — jp3c05332_si_001.pdf [file jp3c05332_si_001.pdf]

Supporting information for:

Morphology and Transport Study of Acid-Base Blend Proton Exchange Membranes by Molecular Simulations: Case of Chitosan/Nafion

Ehsan Hemmasi[1], Mahdi Tohidian[1], Hesam Makki[*2]

[1] *Department of Polymer and Color Engineering, Amirkabir University of Technology, 424 Hafez Ave., Tehran, Iran.*
[2] *Department of Chemistry and Materials Innovation Factory, University of Liverpool, Liverpool L69 7ZD, U.K.*

*Corresponding Author:
Hesam Makki
Email: hmakki@liverpool.ac.uk

Contents

1- Atomistic simulation details……….…………………………………………………S1

2- Coarse-grained (CG) Parameters……………………………………………………S2

3- Validation of models…………………….……………………….……………………S3

3-1- Equilibration time………………………………..…………………………………..S3

3-2- System size effect………………………………………………………………………...S3

3-3- Density and diffusion coefficients………….…………………………………………S3

4- Whole box RDFs…………………………………….………………………………..S4

5- Snapshots of hydrated membranes………………………………………………...S5

6- MSD plots……………………………………………………………………………...S6

7- snapshots of blend membranes at their maximum water content………………...S7

8- Universal plot of water diffusion coefficients versus *PLD*…………………………S8

1- Atomistic simulation details

All-atom (AA) MD simulations on bulk chitosan and Nafion comprising of 20 chains were separately performed to achieve a starting point for mapping the coarse-grained structures. CHARMM 36 force field was adopted for atomistic simulations.[1] Chitosan and Nafion chains were randomly inserted in 20 nm × 20 nm × 20 nm simulation boxes and followed by energy minimization and a NVT run at 300 K for 100 ps (1 fs time step) and NPT run at 1 bar for 60 ns (2 fs time step), subsequently, to ensure the systems equilibration. V-rescale thermostat and Parrinello-Rahman barostat were used to control the temperature and pressure, respectively. See Table S3 for comparison between equilibrated atomistic and experimental densities of bulk chitosan and Nafion.

2- Coarse-grained (CG) Parameters

After reaching the equilibration in AA simulations, we mapped the relaxed AA configuration to coarse-grained (CG) representation based on the Martini methodology. According to the Martini approach, interactions between connected beads (each CG bead represents the center of geometry of the selected atoms belonging to the bead), i.e., harmonic potentials for bonds and angles, are defined by means of equations S2 and S3. For Nafion, we calculated CG bonded interaction parameters by using the distance and angle distributions from the AA simulation as the target properties. To this end, the AA probability distributions of bonds length and angles by averaging over the last 5 ns of the equilibrated AA trajectories were converted to CG bond and angle potentials through AA bond and angle average of Boltzmann's factor (equation S1).[2,3] Then, the CG bond and angle potentials were fitted to the harmonic potential equations (equations S2 and S3) to determine the CG bonded parameters. Figure S1 shows the CG representation of Nafion (and chitosan) and Table S1 provides the calculated parameters. For chitosan, we acquired CG bonded interaction parameters directly from the study of Xu et al.[4]

$$U = -kTln(p) \tag{S1}$$

Where U is the potential, P is the probability, k is Boltzmann constant and T is the absolute temperature.

$$U_{bond} = \frac{1}{2}K_{bond}(l - l_0)^2 \tag{S2}$$

$$U_{angle} = \frac{1}{2}K_{angle}(\cos(\theta) - \cos(\theta_0))^2 \tag{S3}$$

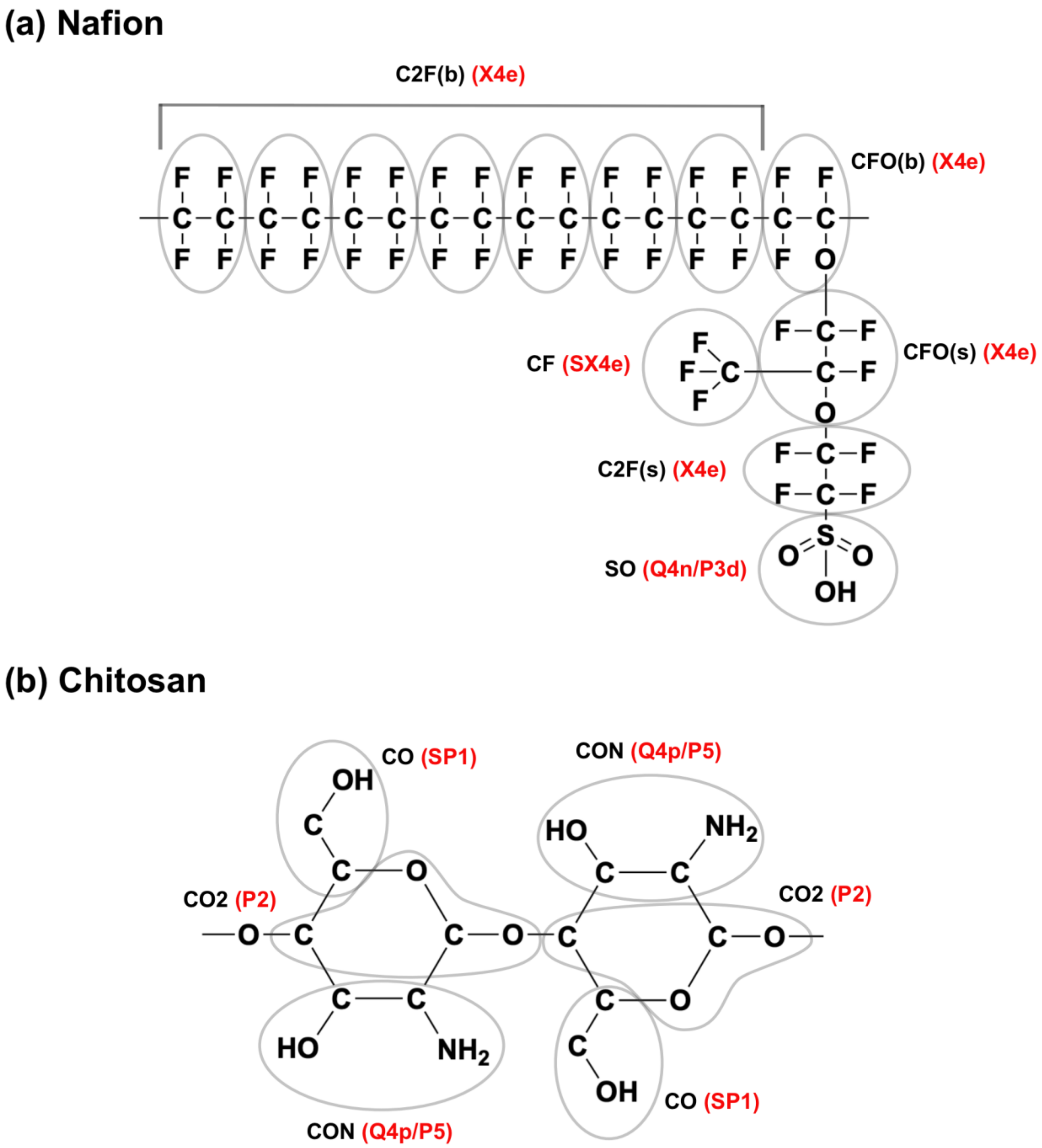

Figure S1. CG representation of Nafion (a) and chitosan (b). Black label devoted to each partition is arbitrary naming and red label in parentheses is bead typing in Martini 3 force field. For Nafion, the SO partition (sulfonic acid group), P3d and Q4n are its bead types in neutral (dry, SO_3H) and ionized state (hydrated, SO_3^-), respectively. For chitosan, the CON partition, P5 and Q4p are its bead types in neutral (amine group, NH_2) and ionized state (protonated amine group, NH_3^+), respectively.

Table S1. Bond and angle parameters of CG Nafion.

Bond	l_0 (nm)	K_{bond} (kj/mol)
C2F(b)-C2F(b)	0.2841	101101
C2F(b)-CFO(b)	0.2581	86280
CFO(b)-CFO(s)	0.3635	38533
CFO(s)-CF	0.2578	75840
CFO(s)-C2F(s)	0.3310	28280
C2F(s)-SO	0.2765	30753
**Angle**	**θ_0 (degree)**	**K_{angle} (kj/mol)**
C2F(b)-C2F(b)-C2F(b)	128.71	543.36
C2F(b)-C2F(b)-CFO(b)	121.45	511.00
C2F(b)-CFO(b)-CFO(s)	92.45	573.30
CFO(b)-CFO(s)-CF	95.00	562.00
CFO(b)-CFO(s)-C2F(s)	143.47	740.80
CF-CFO(s)-C2F(s)	85.20	483.20
CFO(s)-C2F(s)-SO	111.49	484.30

Table S2. Details of atomic partitions.

Atomic partitions	Bead type	Charge	Mass (amu)
**C2F(b) & C2F(s)**	X4e	0	100
**CFO(b) & CFO(s)**	X4e	0	85
**CF**	SX4e	0	69
**SO-** neutral/ionized	P3d/Q4n	0/-1	81/80
**CO2**	P2	0	59
**CO**	SP1	0	44
**CON-** neutral/ionized	P5/Q4p	0/+1	59/60
**water**	W	0	72
**Hydronium ion**	Q4p	+1	19
**Counter ion**	Q1	-1	17
**Sulfate ion (SO_4^{2-})**	D	-2	96
**Methanol**	P3	0	32

We performed CG simulations on both bulk 20-chains chitosan and Nafion with similar equilibration procedure mentioned in the main article, section 2-3, for a brief comparison between AA and CG bulk densities and radius of gyrations (R_g), and also examine the accuracy of our CG parametrization (Table S3). Moreover, Figure S2 shows some examples of AA and CG bond length and angle value distributions for bulk Nafion system. As shown, a relatively good match between AA and CG distributions is achieved.

Table S3. Comparison of densities and radius of gyrations of bulk chitosan and Nafion at AA and CG levels. Values are measured at 300 K.

	**Density (kg/m^3)**			**Radius of gyration (nm)**	
	**Experimental**[5,6]	**Atomistic**	**Coarse-grained**	**Atomistic**	**Coarse-grained**
**Chitosan**	1400	1293	1343	2.509	2.471
**Nafion**	2100	2067	2311	2.858	2.718

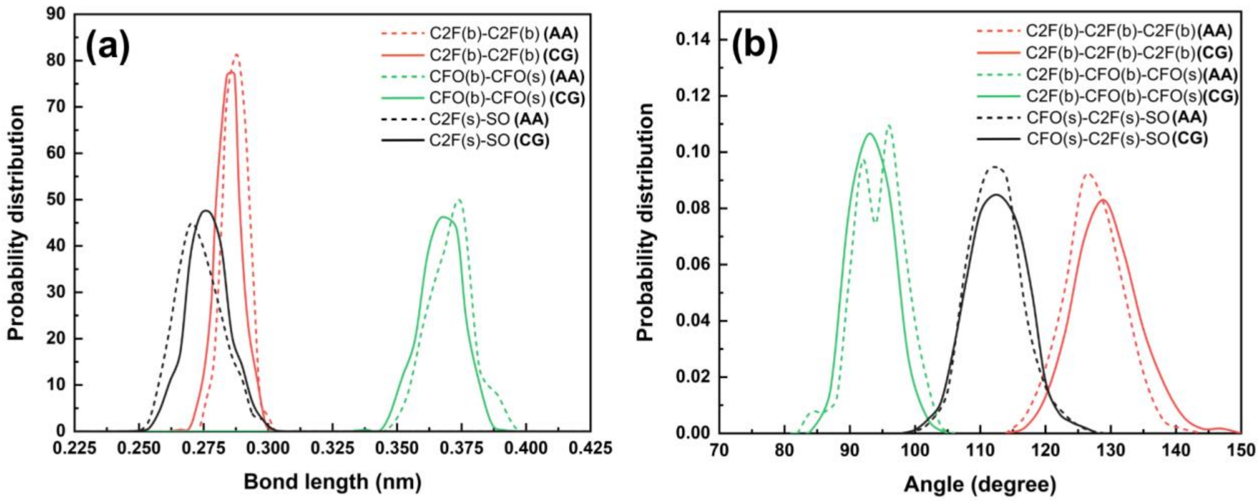

Figure S2. Examples of probability distributions of (a) bond lengths, and of (b) angles for 20-chains bulk Nafion system from AA and CG simulations.

3- Validation of models

3-1- Equilibration time

To evaluate whether the simulation time of our CG simulations were appropriate to achieve equilibrated membrane systems, we followed variations of the squared radius of gyration autocorrelation function (ACF) and density over simulation time in the last step of equilibration procedure (mentioned in section 2-3 in the article, 60 ns of NPT run at P = 1 bar and T = 300 K). Figures S3a and S3b display the squared radius of gyration ACF and density of Nafion system at

WC = 5%, respectively. It can be seen that the squared radius of gyration ACF converges into zero (the dotted line in Figure S3a) before 12 ns. The variation of density also becomes independent of time after 10 ns. Figures S3c and S3d show the squared radius of gyration ACF and density of blend-25 system at WC = 10%, respectively. As shown, the squared radius of gyration ACF of Nafion and chitosan converge into the zero (the dotted line in Figure S3c) before 15 ns. The variation of density also becomes independent of time after 5 ns. With all that said, we can be sure that the simulation time (60 ns) is long enough to produce equilibrated membrane systems.

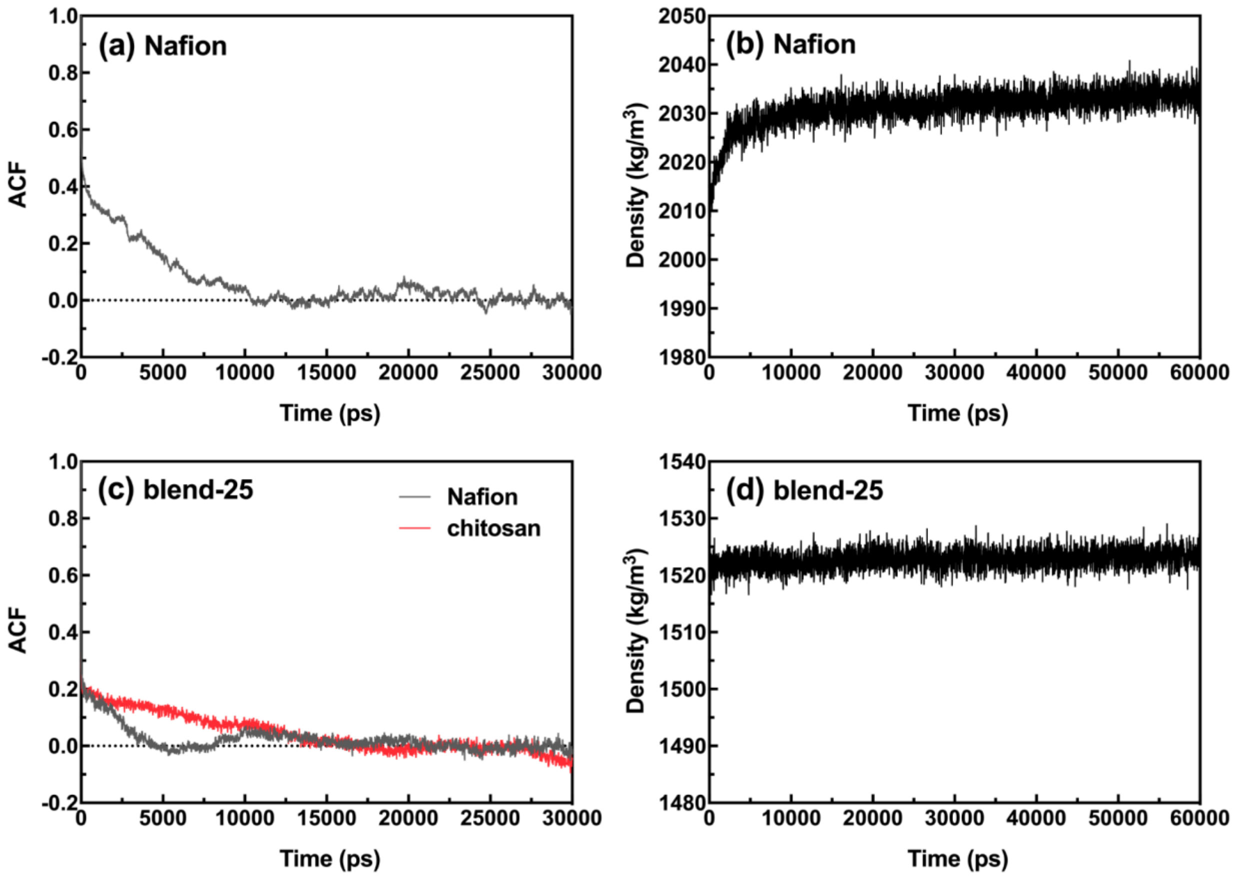

Figure S3. The squared radius of gyration autocorrelation function (ACF) and density over simulation time for Nafion and blend-25 systems at WC = 5% and WC = 10%, respectively.

3-2- System size effect

We assessed possible system size effect on density, *PLD*, and $g_{Q4n\text{-}W}$ of both Nafion and blend-25 systems at WC = 20%. As can be seen from Figure S4, for Nafion system, no changes in the specified characteristics occurs above 60 Nafion chains contained systems. For blend-25 system also, the specified characteristics become independent of the number of Nafion chains above 11. Therefore, the results of our CG simulations for both membrane systems, i.e., 100 Nafion chains

system (Nafion) and 15 Nafion chains system (105 chitosan chains, blend-25), are independent of the system size.

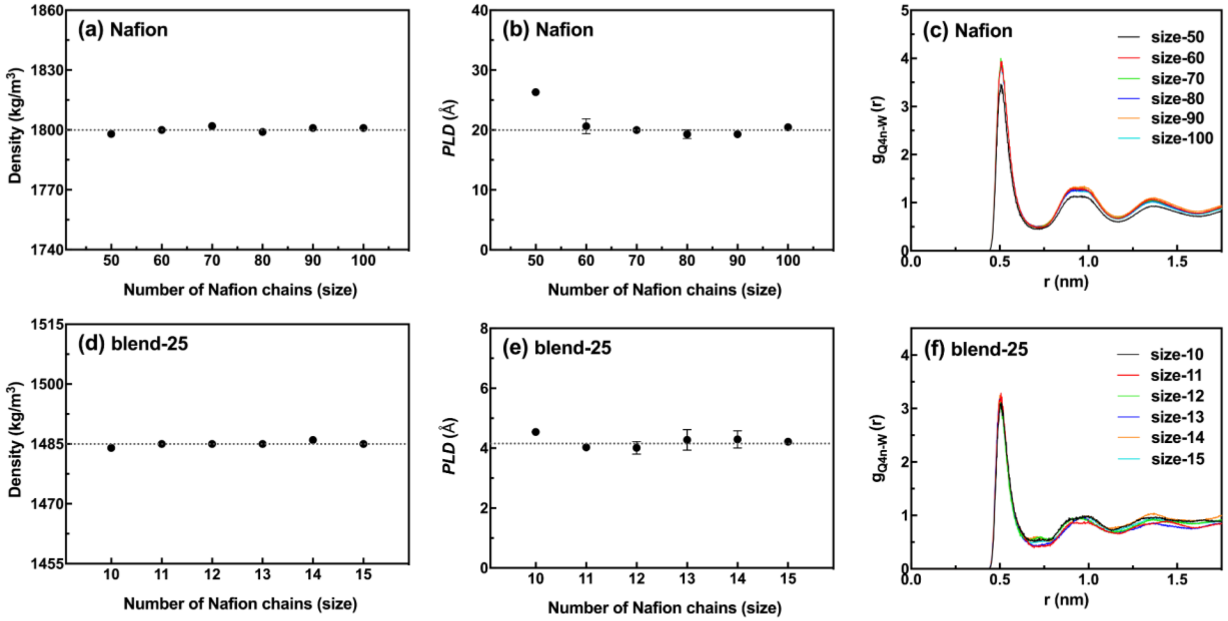

Figure S4. The density, *PLD*, and $g_{Q4n\text{-}W}$ characteristics for different system sizes of Nafion and blend-25 systems at WC = 20%.

Additionally, we performed water cluster analysis for Nafion system containing 80 Nafion chains (size-80) at WC = 5% through 35%, and compared the normalized cluster size results with the original Nafion system in the article (100-chains Nafion system (size-100), Figure 4a in the article). The comparison between these two systems is shown in Figure S5. As seen, no significant difference can be observed between these two systems, and independency of our results from the system size is still valid.

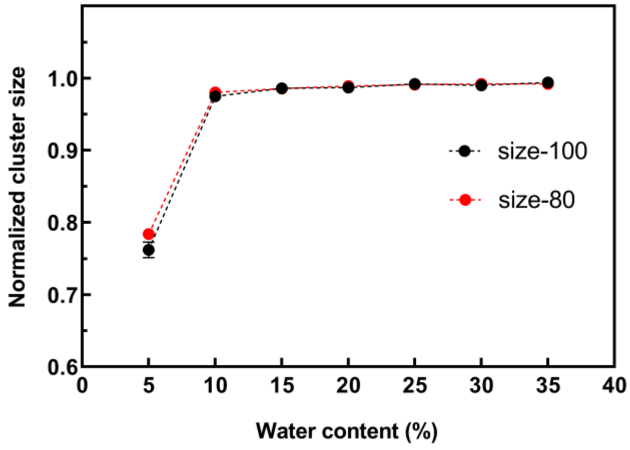

Figure S5. Normalized cluster size versus water content for size-100 and size-80 Nafion systems.

3-3- Density and diffusion coefficients

The density, water diffusion coefficient, and hydronium ion diffusion coefficient of Nafion system are plotted as a function of hydration level, λ, in Figure S6. Hydration levels for our hydrated Nafion system vary between 3.21 to 22.5, corresponding to WC = 5% to WC = 35% (Table1 in the article). For density (Figure S6a), a good agreement with the experimental data obtained from a fitting equation[7] can be seen. Moreover, comparable trends for water and hydronium diffusion coefficients with atomistic diffusion data are also achieved.[8,9] It should be noted that CG diffusion data must not be compared quantitively by atomistic data due to the smoothness of the CG potential landscape, and any quantitative match must be accidental. Atomistic data are only presented to show the increasing diffusion trends with increasing hydration level.

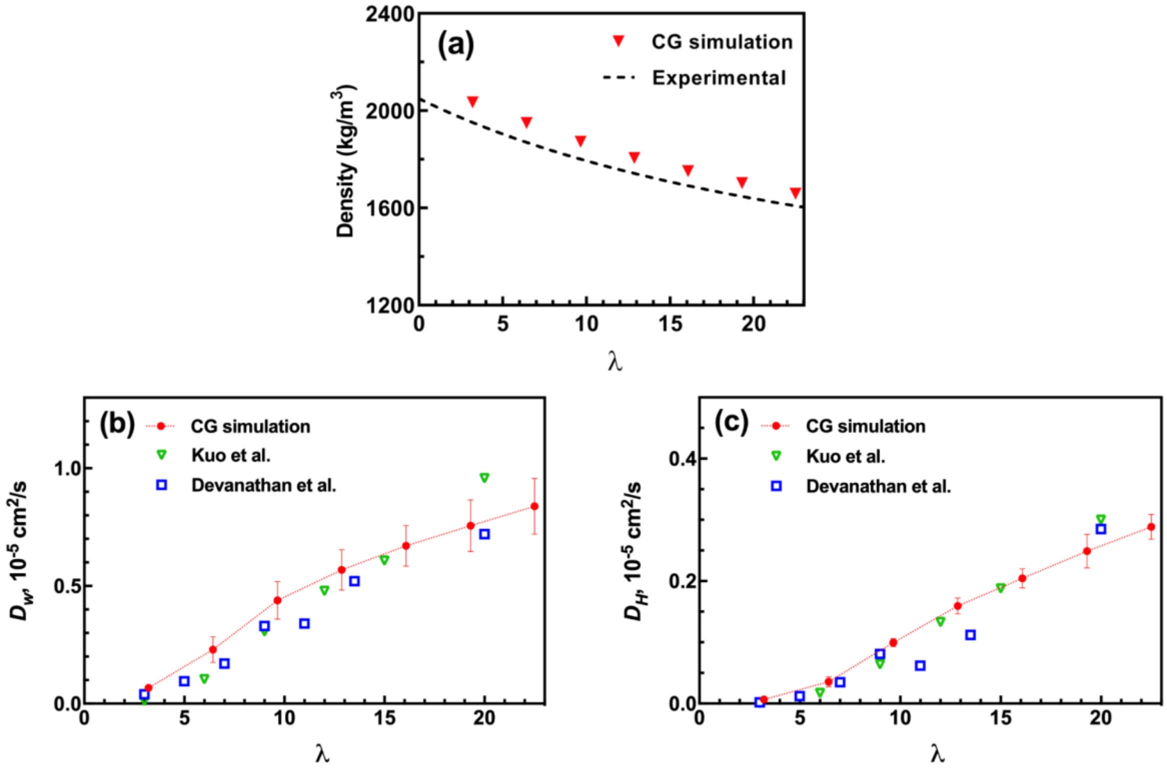

Figure S6. Density, water diffusion coefficient, and hydronium ion diffusion coefficient of Nafion system as a function of hydration level (λ), and corresponding experimental and atomistic simulation data (just for qualitative comparison of diffusion trends).

4- Whole box RDFs

Extended some RDFs for Nafion system to whole box dimensions are provided in Figure S7.

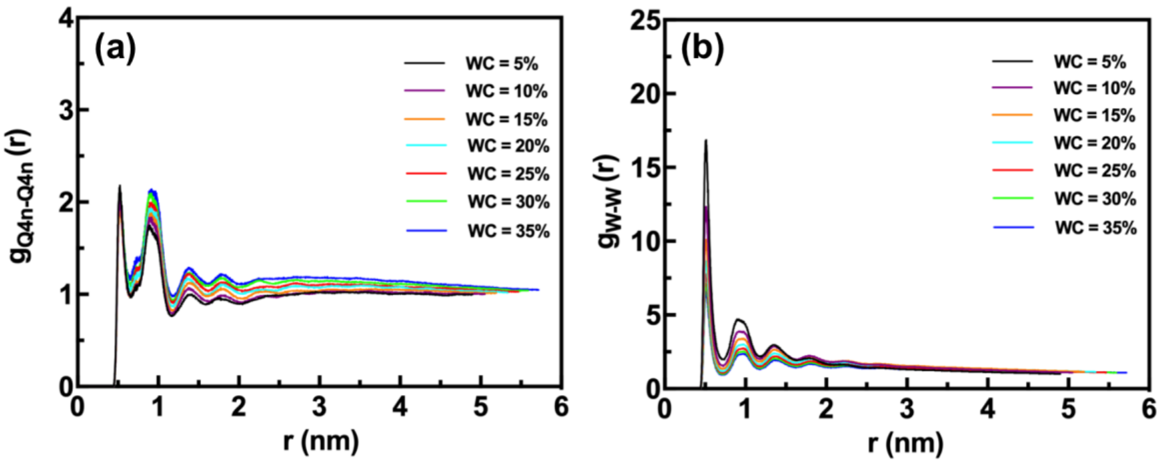

Figure S7. Whole box RDF of (a) Nafion sulfonate-sulfonate beads ($g_{Q4n-Q4n}$) and (b) water-water beads (g_{W-W}) for Nafion system as a function of water content (WC).

5- Snapshots of hydrated membranes

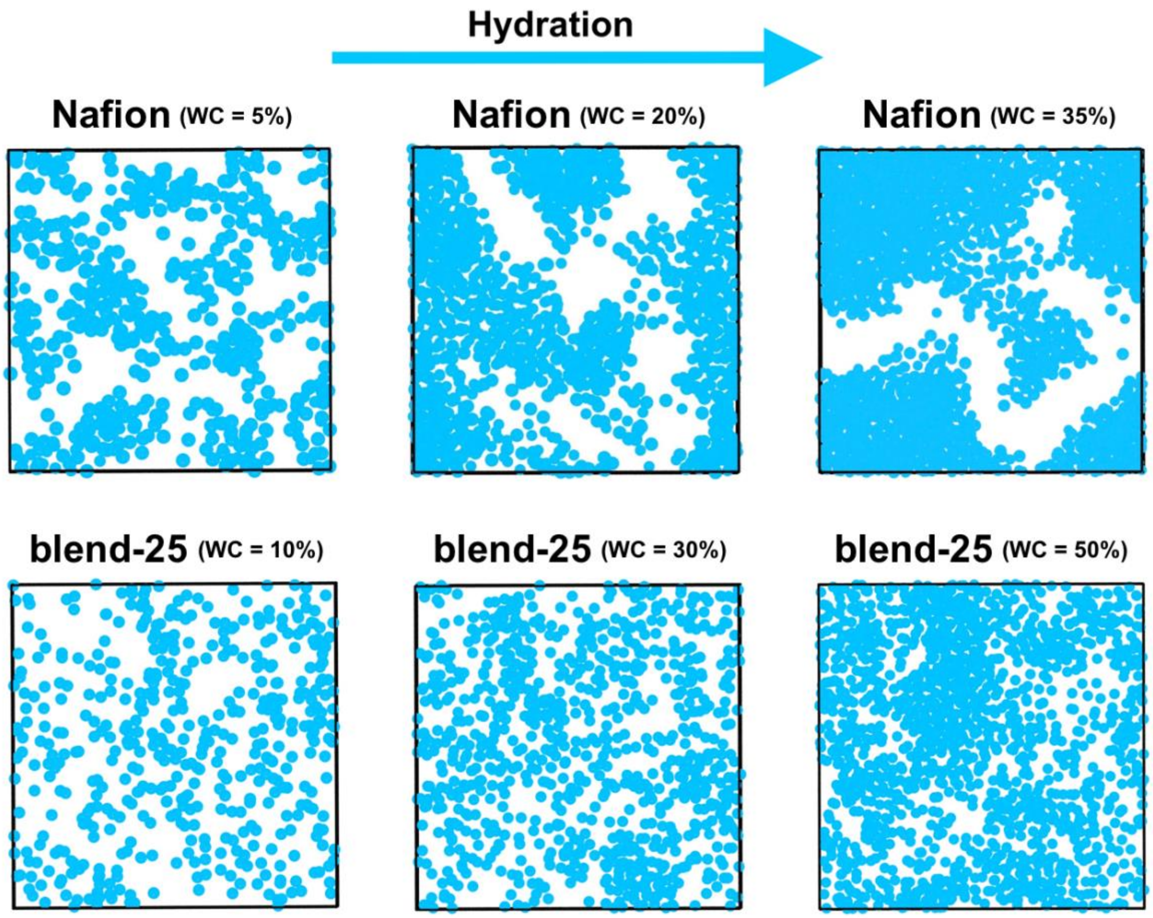

Figure S8. 2D snapshots of Nafion and blend-25 systems upon hydration. Water beads (and hydronium beads if exist) are colored in blue, and polymer chains are excluded from the snapshots (white areas)

6- MSD plots

Figure S9 shows the MSD plots on a log-log scale, from which diffusion coefficients were calculated in the section 3-3 of the article.

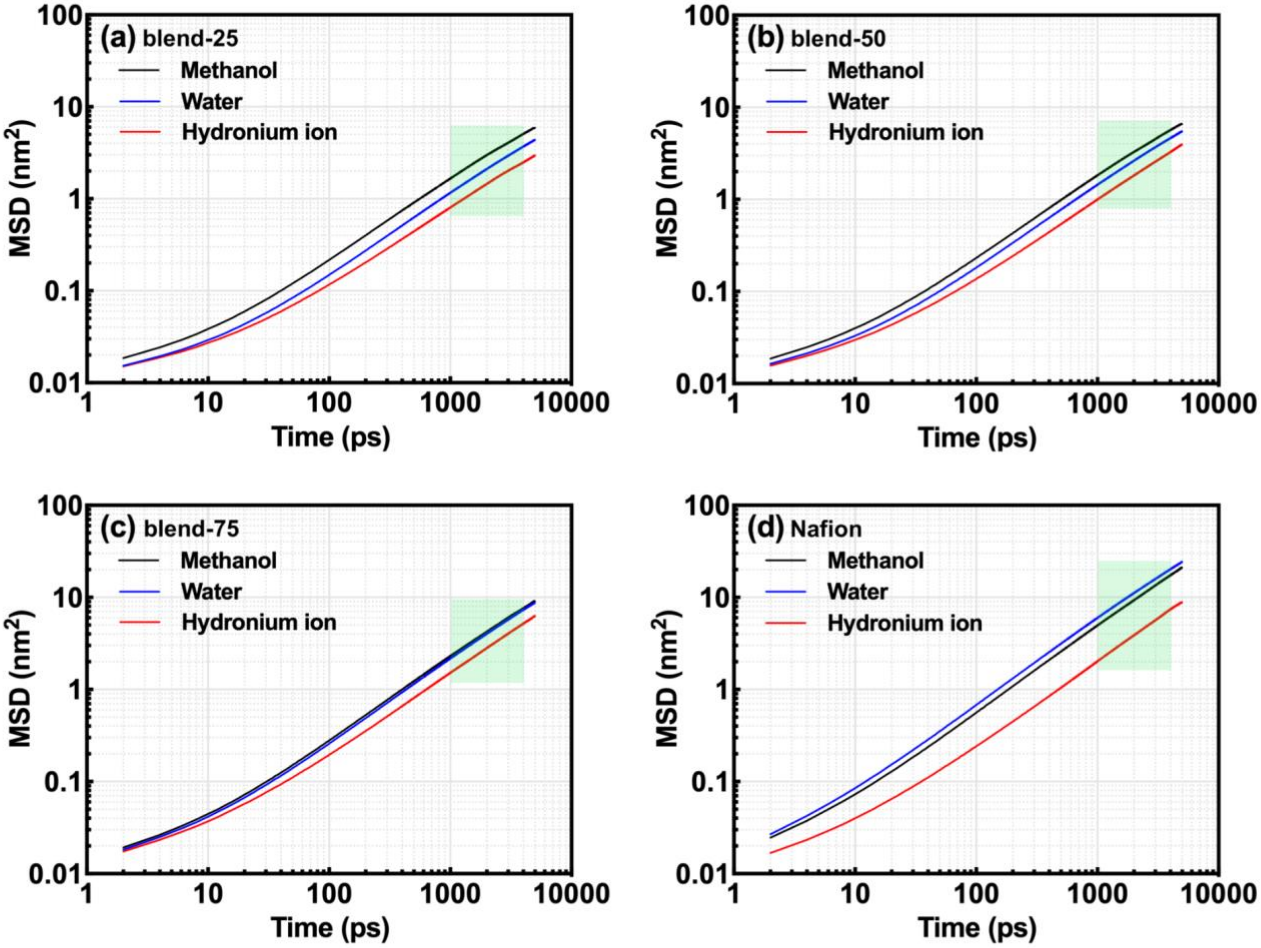

Figure S9. MSDs of methanol, water, and hydronium ion as a function of time plotted on a log-log scale for (a) blend-25, (b) blend-50, (c) blend-75, and (d) Nafion systems. the highlighted green square shows the calculated region for diffusion coefficients.

7- Snapshots of blend membranes at their maximum water content

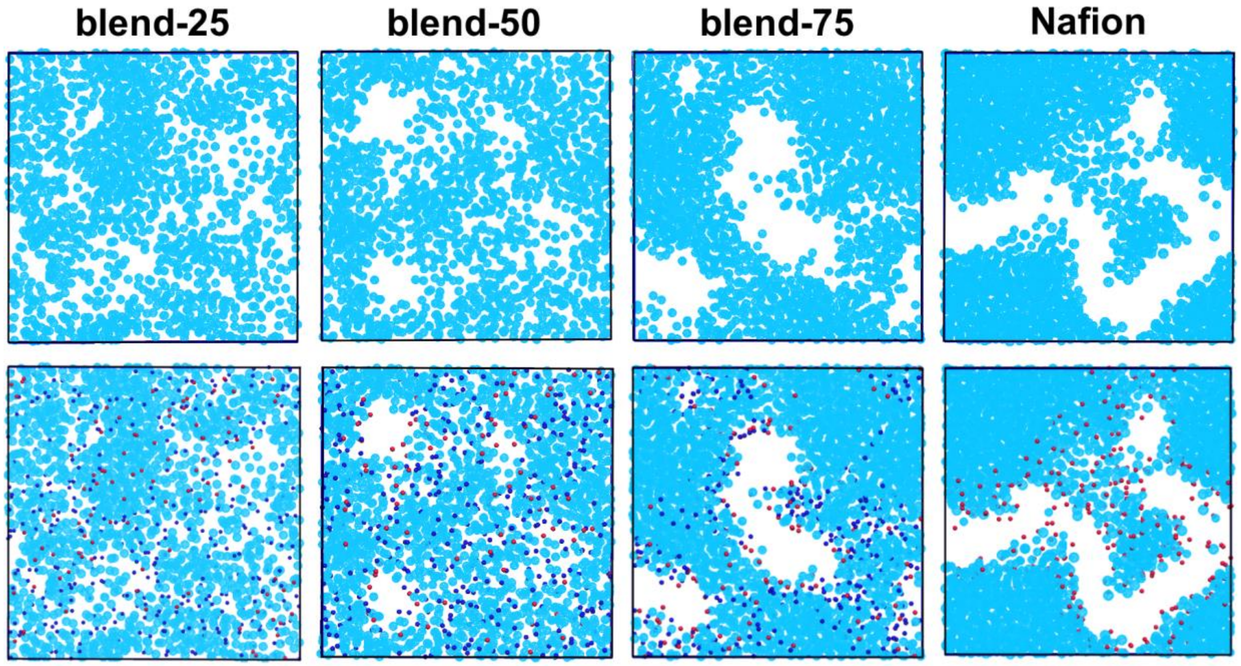

Figure S10. 2D snapshots of blend systems at their maximum water contents with increment of Nafion content. Water beads (and hydronium beads if exist) are colored in blue, and polymer chains are excluded from the snapshots (white areas). Lower panel shows the same snapshots as upper panel with charges distribution included. Dark blue and red dots show positive (of chitosan) and negative (of Nafion) charges, respectively.

8- Universal plot of water diffusion coefficients versus *PLD*

All water diffusion coefficients in our study are depicted as a function of *PLD* and presented in Figure S11. Water diffusion coefficients associated to Nafion system are in blue, blend-25 in red, blend-50 in green, and blend-75 in purple. The diffusion-*PLD* correlation is shown by a dashed straight line.

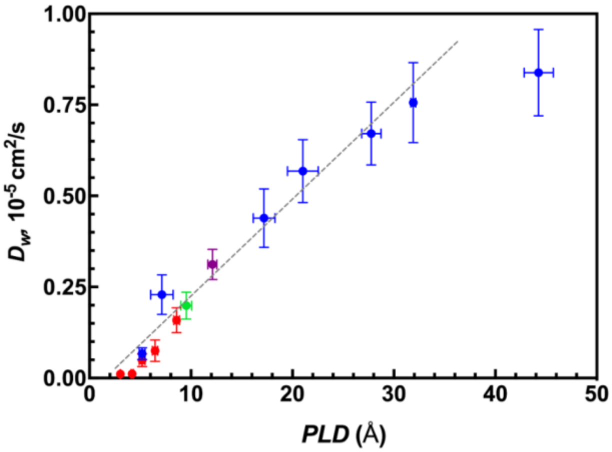

Figure S11. Universal plot of water diffusion coefficients versus *PLD*. Water diffusion coefficients associated to Nafion system are in blue, blend-25 in red, blend-50 in green, and blend-75 in purple.

References:

(1) Best, R. B.; Zhu, X.; Shim, J.; Lopes, P. E. M.; Mittal, J.; Feig, M.; MacKerell, A. D. Optimization of the Additive CHARMM All-Atom Protein Force Field Targeting Improved Sampling of the Backbone φ, ψ and Side-Chain X1 and X2 Dihedral Angles. *J. Chem. Theory Comput.* **2012**, *8* (9), 3257–3273.

(2) Morohoshi, K.; Hayashi, T. Modeling and Simulation for Fuel Cell Polymer Electrolyte Membrane. *Polymers (Basel).* **2013**, *5* (1), 56–76.

(3) Ghermezcheshme, H.; Makki, H.; Mohseni, M.; Ebrahimi, M.; De With, G. MARTINI-Based Simulation Method for Step-Growth Polymerization and Its Analysis by Size Exclusion Characterization: A Case Study of Cross-Linked Polyurethane. *Phys. Chem. Chem. Phys.* **2019**, *21* (38), 21603–21614.

(4) Xu, H.; Matysiak, S. Effect of PH on Chitosan Hydrogel Polymer Network Structure. *Chem. Commun.* **2017**, *53* (53), 7373–7376.

(5) Ma, J.; Zhang, M.; Wu, H.; Yin, X.; Chen, J.; Jiang, Z. Mussel-Inspired Fabrication of Structurally Stable Chitosan/Polyacrylonitrile Composite Membrane for Pervaporation Dehydration. *J. Memb. Sci.* **2010**, *348* (1–2), 150–159.

(6) Kusoglu, A.; Weber, A. Z. New Insights into Perfluorinated Sulfonic-Acid Ionomers. *Chem. Rev.* **2017**, *117* (3), 987–1104.

(7) Weber, A. Z.; Newman, J. Transport in Polymer-Electrolyte Membranes. *J. Electrochem. Soc.* **2004**, *151* (2), A311.

(8) Devanathan, R.; Venkatnathan, A.; Dupuis, M. Atomistic Simulation of Nafion Membrane. 2. Dynamics of Water Molecules and Hydronium Ions. *J. Phys. Chem. B* **2007**, *111* (45), 13006–13013.

(9) Kuo, A. T.; Shinoda, W.; Okazaki, S. Molecular Dynamics Study of the Morphology of Hydrated Perfluorosulfonic Acid Polymer Membranes. *J. Phys. Chem. C* **2016**, *120* (45), 25832–25842.
